# What do students expect from teaching assistants (TAs)? Considerations of gender, race, and international status

**DOI:** 10.1371/journal.pone.0334904

**Published:** 2025-10-30

**Authors:** Denise Wilson, Neha Kardam

**Affiliations:** Department of Electrical and Computer Engineering, University of Washington, Seattle, Washington, United States of America; Touro University, UNITED STATES OF AMERICA

## Abstract

Teaching Assistants (TAs) make important contributions to STEM teaching in higher education. While TAs often play both peer and authority figure roles, however, relatively little is known about exactly what students expect from TAs. To fill this gap, the first major goal of this study was to comprehensively understand these expectations from a large population of undergraduate engineering students. In addition, this study sought to understand how these expectations vary with students’ gender, race/ethnicity, and international status during distinct time periods associated with the recent COVID-19 pandemic (pre-COVID, during COVID, and post COVID). Student expectations were measured via a short-answer survey question in a cross-sectional dataset at a single, large institution comprised of sophomore to senior level students (**n* *= 1,678) enrolled in engineering courses between 2016 and 2023. Thematic analyses were used to analyze student expectations and statistical, quantitative techniques were used to identify demographic differences. While no single majority theme emerged, many (42.9%) students thought that interactions were most important for TAs to emphasize while 37.6% believed TA preparation to be most important. A smaller but noteworthy percentage (7.61%) of students expected TAs to be caring and hospitable. Significant differences emerged in different time periods and across students’ race/ethnicity, international status, and gender. The results of this study indicate that students have a wide range of expectations of TAs and that these expectations are different for different times and classroom conditions. The results of this study can directly inform TA professional development as well as faculty guidance and supervision of TAs.

## Introduction

Engineering has a diversity problem that has persisted for a very long time. For example, in 2022, women accounted for less than a quarter (24.2%) of engineering bachelor’s degrees in the U.S., marking a significant increase from the 17.8% recorded in 2010 [1], but the number of women engineers in the workforce has only risen from 10.5% in 2012 to 13.7% in 2021 [2]. The underrepresentation of women is particularly pronounced in mechanical, electrical, and computer engineering, with women representing only 9%, 10%, and 12% of working engineers in these respective fields [3]. For underrepresented minorities, the statistics are even more dismal. Bachelor’s degrees in engineering awarded to Black or African American individuals have risen only slightly from 4.5% in 2010 of all such degrees to 4.7% in 2021 [1]. Hispanics now earn about 13.6% of bachelor’s degrees in engineering up from 7.0% in 2010 [1] but the Hispanic population in the U.S. has proportionately increased over the same time period from a little over 35 million individuals (12.2% of the U.S. population) to over 62 million (18.8% of the general population) [4].

The underrepresentation problem is not just about how few women and underrepresented minorities (URMs) pursue engineering degrees. The leaky pipeline in engineering and other STEM fields continues after the initial choice of major, leading to drop-out rates among women and URMs in college that are significantly higher than majority populations [5,6]. Faculty and other instructors have a valuable opportunity to reduce these exits. Yet, by the very nature of the feedback instructors receive regarding their teaching, they may be inadvertently contributing to drop-out. 94.2% of colleges use student evaluations of teaching (SETs) to provide feedback to instructors about teaching performance [7]. In addition to being biased against underrepresented faculty and being poorly correlated to teaching effectiveness [8,9], SETs fail to adequately represent underrepresented students because, in large part, they do not disclose demographics. As a result, SETs can misdirect teaching improvements. When students provide suggestions for improved teaching via short answer questions on SETs, faculty and other instructors who act on these suggestions may inadvertently be favoring the most frequently cited issues and therefore give priority to majority group(s) over underrepresented students. This study seeks to better understand whether such differences between majority and minority groups do in fact exist. However, rather than situate this question in the relatively crowded space of faculty support literature, our study instead focuses on teaching assistants (TAs) who are understudied compared to faculty but nevertheless play an important and distinct role in engineering education. Knowing whether and where demographic differences in student expectations of instructors exist is essential to nourishing diversity in engineering learning communities by including all voices, whether over- or under- represented, in efforts to improve teaching.

### The importance of TA support

Postsecondary Teaching Assistants (hereafter referred to as TAs) play a significant role in higher education in the United States. Not surprisingly, colleges, universities, and professional schools employ the highest level of TAs at 115,990 or 3.84% of their overall workforce [10]. Among state or government owned institutions, the proportion is even higher. 77,880 individuals are employed as TAs, making up 4.64% of the total workforce of 1,679,110 [11]. TAs are especially prevalent in science and engineering at research universities with one survey indicating that 91% of all biology labs and 70% of life and physical science labs are taught by TAs rather than faculty [12]. Since most degrees in engineering are awarded at research universities [1], it stands to reason that most undergraduates in these disciplines receive a significant amount of their education from TAs and in many classes, TAs are more visible and accessible to them than faculty. Yet, despite the large numbers of TAs in science and engineering programs, “this group of teachers is almost invisible in the academic machinery that drives educational programs at large universities” [13, p. 31]. Despite their invisibility, TAs nevertheless act as agents assigned to carry out the teaching and learning objectives of the faculty member. As a result, TAs are often the “first line of defense” [14, p. 8] in undergraduate instruction. What TAs do affects the perceived quality of the overall curriculum in the department [14], student engagement [15], the faculty member’s teaching [14,16], student enjoyment and satisfaction [16], content knowledge among students [17], and student comfort and questioning behaviors in the classroom [18].

Unlike faculty who are viewed as distant and formal sources of structure and knowledge, TAs act as both a social and an academic support to students. Kendall and Schussler [19] found that “regardless of type of class, professors were perceived as being confident, in control, organized, experienced, knowledgeable, distant, formal, strict, hard, boring, and respected.” while TAs were perceived as “…uncertain, hesitant, nervous, relaxed, laid-back, engaging, interactive, relatable, understanding, and able to personalize teaching” [19, p. 187]. These results are not surprising considering that compared to faculty, TAs are more likely to be similar in social status and age to the undergraduates they teach, thus leading students to connect with them as both a peer and a person of authority. Given these role differences, however, the faculty support literature should be interpreted cautiously when applying it to TA professional development and as importantly, the instructional support of TAs should be studied separately from that provided by faculty. Recognizing this and in response to the limited research on TA support in the literature, this study sought to better understand what engineering students’ expectations of TAs are via the following research question:

#### Research Question #1 (RQ1): What do undergraduates expect from TAs?

This research question is explored using an open-ended, short answer survey question that does not restrict students to a preconceived framework for effective TA teaching. The answer to this question across a large population of students can provide meaningful recommendations for TA teaching and professional development.

### The importance of student demographics

A limited body of research has investigated if instructional support (whether perceived or observed) varies with students’ gender, race, ethnicity, or country of origin. For example, a study of over 650 students in middle and high school showed that perceptions of instructor care reported by female students were significantly higher than those reported by male students [20]. However, another K-12 study contradicted these results and found no such differences in student perceptions of instructor support [21]. This absence of gender differences has also emerged in higher education studies. In studying 24 classrooms across eight major departments at a major university, Brady & Eisler [22] found that neither actual classroom participation nor student perceptions of classroom support varied across gender. Similarly, no differences in how instructors interacted with male and female students were observed [22].

Introducing race and ethnicity into examining gender in instructional support provides more nuanced insight into the role of demographics in perceptions of such support. In a recent study involving interviews of over 200 male and female seniors in STEM majors, Rainey et al. [23] found that female students thought that their instructors cared about and supported their learning more so than male students reported such care. But this result was only true for white female students. In contrast, women of color reported much lower perceptions of care from instructors than white female students and all male students reported, regardless of race [23]. But in high school, both male and female Black students have reported that teachers offer less support (in terms of caring) to them than their white peers report, regardless of students’ socioeconomic status or schools’ socioeconomic context or diversity [24].

Country of origin also affects how students view their instructors. Certain international students struggle with low perceptions of instructional support. For example, a narrative study by Glass et al. [25] showed that international students who have low financial resources and low academic preparedness reported no positive interactions with faculty at all, indicating a clear deficit in instructional support for their studies. International students who do not come from Western or English-speaking countries also reported greater discrimination and negative experiences, including reduced or hostile instructional support, compared to what their peers who arrive from Western and English-speaking countries report [26].

In addition to directly studying instructional support in and of itself, another body of research has also uncovered demographic differences in the impacts of instructional support. In both K-12 and in higher education, research has shown that both cross-sectional and longitudinal connections between student perceptions of instructional support and student academic outcomes vary across gender, race, or country of origin. For instance, in a study of over 600 undergraduates in engineering at a major public university, female students paid more attention as a result of increased support from faculty but the same was not true for male students [27]. Yet, when instructors provided support through greater active learning opportunities, male students in biology courses engaged more (as measured by participation) and exhibited greater science self-efficacy than female students in these settings [28]. At K-12 levels, male students also appear to benefit more from instructional support. In a longitudinal study of K-12 (grades 4–11) students, teacher/instructor support predicted greater school satisfaction for male students than for female students [29]. Males and females also respond differently to different types of instructional support. For instance, structured learning activities have been shown to influence engagement more for male students while support for the learning process itself is more important to impacting the engagement of female students [30].

While these studies do not make for a comprehensive understanding of the ways in which different types of students perceive instructional support nor how instructional support affects them, they nevertheless provide sufficient evidence that student demographics do indeed matter when considering who, how, when, and where to offer such support. In order to improve teaching and academic support practices, however, it is important to understand not only what these demographic differences are but also whether they are a result of instructors treating students differently, of students interpreting what instructors do differently, or of students having different expectations of what instructors should be doing. *This study* seeks to further explore the last of these three factors by specifically exploring demographic differences in what student expectations for what instructional support (from TAs) should look like. This interest in demographic differences drives the second and last primary research question in our study:

#### Research Question #2 (RQ2): Does a student’s race, gender, or international status affect their expectations of TAs?

Uncovering and considering demographic differences in preferences for instructional support is essential to facilitating inclusive classrooms, laboratories, and other learning environments in engineering. Investigating whether such demographic differences exist in what students expect from TAs and whether those preferences are different at different times and classroom conditions is essential to informing TA professional development and mentor/supervisor support. Such insight is also important to understanding whether student preferences should be measured as a diagnostic tool for adjusting instructional practice from term-to-term, year-to-year, or over longer time periods.

### Conceptual model

*This study* focuses on instructional support provided by TAs rather than faculty both because of the scarcity of existing studies that focus on TAs and the dual roles that TAs are likely to play in students’ education. TAs are often of similar age and provide direct support to students, thereby placing them in a peer support role. Concurrently, however, they also hold a level of authority in delivering lessons, leading labs, providing individual guidance, and maintaining other similar responsibilities, putting them in an authority figure role. These dual roles are likely to create different expectations from students when compared to what students expect from faculty.

**Fig 1:**
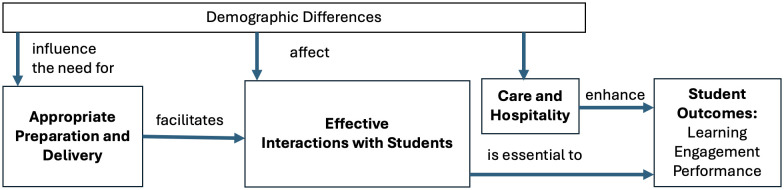
Conceptual model for this study.

The conceptual model that guided this study (Fig 1) made room for three broad themes in the analysis of preferences for TA support expressed by students. The model posits that preparation facilitates effective TA-student interactions in a similar manner to how faculty preparation has been demonstrated to facilitate effective faculty-student interactions [31]. Based on a meta-analysis of factors that influence faculty-student interactions [31], preparation includes professional factors (appropriate content knowledge and skills); communication factors (communication and delivery skills as well as effective use of technology); management factors (classroom/lab rules and access to support); and educational factors (educational methods and resources). The effective faculty-student interactions that result from high-quality preparation, in turn, are essential to support academic outcomes and overall success including engagement and grades [32]. The conceptual model used in this study posits that these same impacts are at work in the TA-student relationship. The care, concern, respect and overall hospitality instructors (including TAs) show for students by instructors is especially important for creating a nourishing and inclusive learning environment [33] which, in turn, enhances how their interactions can lead to positive academic outcomes.

These three themes (preparation, interactions, and hospitality) were chosen to highlight student expectations of TAs, independent of their use of language or underlying knowledge of effective pedagogy. This approach, in turn, is likely to lead to more direct and actionable recommendations for TA practice in engineering and related STEM fields. These three themes also guided the analysis of the first research question (RQ1) associated with this study (*What do undergraduates expect from TAs?)*.

Furthermore, the degree to which students benefit from and perceive the importance of these three different elements of TA instruction is likely to vary depending on the past experiences and pre-entry attributes that students bring to the table. In this study, such variability is examined through a lens of internal diversity (race and gender demographics, discussed in the Importance of Student Demographics section of this paper) and pre-entry characteristics to answer our second research question (RQ2): *Does a student’s race, gender, or international status affect their expectations of TAs?.*

## Materials and methods

This study is part of a larger, single-institution research project which used a survey to investigate the connections between various sources of instructional support and multiple dimensions of course-level engagement. The survey included multiple demographic items as well as Likert-scale items to measure engagement, belonging, task value, and self-efficacy, and five short answer questions. Three short answer questions asked students to comment on their expectations for peer support, one question inquired about faculty support, and one inquired about TA support. Race, gender, parents’ education, GPA, family income, and international status data were used to analyze data in this study alongside student responses to the short answer TA support question:

“What one action can your TAs at <this institution> take to best support you in your classes (please be as specific as possible)?”

This question was posed to students in traditional classroom settings prior to the COVID-19 pandemic (2016–2019), to students taught using emergency remote teaching (ERT) during the peak of the COVID-19 pandemic (2020–2021), and to students enrolled in courses after the peak of the pandemic had passed (post-COVID, 2022–2023). Patterns of preferences for TA support as well as differences among these three time periods and across student gender, race, ethnicity, and country of origin (i.e., international student status) were studied.

### Participant demographics

Across all three time periods studied, a total of 1,678 students completed the short answer question on the survey associated with this study. A detailed breakdown of student demographics is provided in Table 1. Most students were male (*n* = 1,241, 74.0%) and either Asian (**n* *= 485, 34.6%) or Non-Hispanic White (**n* *= 605, 43.2%). Most student respondents were US citizens or permanent residents (**n* *= 1,400, 83.4%). Those races where representation was less than ten individuals in the entire dataset including Native American, Pacific Islander, and most mixed races were combined into a single category labeled “Other URM.” One mixed race (Asian/White) had substantial sample size and was retained as a separate group (*n* = 78, 5.57%). Although Latino is an ethnicity rather than race and refers to an individual whose origin is Latin America or the Caribbean, it was listed as part of a combined race/ethnicity question in this survey with an option for students to select all that apply among one choice for ethnicity (Latino) and multiple choices for race. Of the 64 students who responded as Latino, none also selected a race or races. This is not ideal but likely reflects that Latino students identify primarily by their ethnicity rather than their race. Further, although male and female are often used to describe both sex (assigned at birth) and gender (social construct), this survey explicitly ask respondents to identify their gender as male, female, or other. A demographic question asking about sex was not included in the survey.

**Table 1:** Demographics of study population*.

	All Time Periods		Pre-COVID		ERT		Post-COVID	
	N	%	N	%	N	%	N	%
Demographic								
All	1678	100	534	31.8	766	45.7	378	22.5
*Gender (among all students/participants)*								
Male	1241	74.0	403	75.5	559	73.0	279	73.8
Female	413	24.6	128	24.0	197	25.7	88	23.3
Other	12	0.720	2	0.370	6	0.780	4	1.06
*Race/Ethnicity (among domestic students/participants)*								
Black	32	2.29	10	2.20	15	2.38	7	2.22
Latino	64	4.57	16	3.52	29	4.60	19	6.01
Other URM	110	7.86	38	8.37	46	7.30	26	8.23
Asian	485	34.6	134	29.5	218	34.6	133	42.1
White	605	43.2	226	49.8	269	42.7	110	34.8
Asian/White	78	5.57	24	5.29	39	6.19	15	4.75
*Country of Origin (among all students/participants)*								
Domestic	1400	83.4	454	85.0	630	82.3	316	83.6
International	265	15.8	79	14.8	130	17.0	56	14.8

*Numbers do not necessarily add up to 100% due to non-responses.

Since nearly all the international students who reported their race were Asian (**n* *= 246, 92.8%), race/ethnicity is reported and analyzed for domestic students only. Some students did not respond to certain demographic questions; therefore, the total number of students in each demographic category does not always add up to the total sample. In addition to gender, race/ethnicity, and country of origin demographics, pre-entry attributes measuring family of origin annual income (across eight ranges from $0-$20k to $150k or more) and father’s level of education (across seven levels ranging from did not finish high school to a doctoral degree) were also measured in this study. Student GPA was also collected as a pre-course measure of knowledge and skills. Statistics associated with these pre-entry characteristics were calculated to explore potential causes of differences in students’ perspectives regarding TA support.

### Course demographics

The 43 courses taught by 31 different faculty and surveyed in mechanical and electrical and computer engineering are summarized in Table 2. 90% of courses in engineering supported by one or more TAs over the time period associated with this study had enrollments over 50 students; 86% of courses surveyed were sophomore or junior level courses; and almost half of the courses were surveyed during the COVID-19 pandemic due to increased interest from instructors in understanding the needs of their students during ERT (remote learning). The maximum enrollment for any of the courses studied was in a mechanical engineering course (dynamics) with an enrollment of 263.

**Table 2:** Courses studied.

Time Period	Participation		Student response rate			
	*n *(%) Surveys	*n* (%) Courses	Overall	Min	Mean	Max
Pre-COVID	754 (32.2)	8 (18.6)	78.2	29.0	73.8	92.7
ERT	1142 (48.7)	27 (62.8)	65.4	9.52	66.1	100
Post-COVID	448 (19.1)	8 (18.6)	56.7	6.67	63.7	100
Total	2344 (100)	43 (100)	67.0	6.67	67.1	100

Eight courses including 32.2% of all surveys were collected before the COVID-19 pandemic took hold in spring of 2020. 27 courses including 48.7% of all surveys were collected during the ERT phase of COVID (i.e., remote learning) between spring 2020 and spring 2021 inclusive, and the remaining eight courses (19.1%) of surveys were collected after classes returned to in-person learning in summer 2021. The student response rate ranged from 7% to 100% across all 43 courses, but the average response rate was 67% of all enrolled students in the courses studied. Low response rates occurred in two classes where extra credit was not offered or where the survey was offered late in the term. Some students completed the survey multiple times; duplicates were removed for this study by retaining only the student’s first response (in time), resulting in 1,678 unique responses.

### TA responsibilities and characteristics

In a vast majority of courses that engineering students take as part of their degree program, TAs lead either one or two 110-minute quiz (recitation) sections every week or one or two 3-hour lab sections every week. For lab sections, lab procedures are provided, but TAs are expected to customize and prepare their own materials to guide students through the labs. For quiz sections, TAs are expected to prepare their own materials in response to faculty guidance. While part of the TA’s role in these quiz sections is to review elements of the professor’s lecture that may be confusing to students, most of the materials TAs prepare and deliver to students are original and expand on or are independent of the instructor’s prepared materials. In addition to leading these sections, TAs are also responsible for holding office hours, remaining responsive to students outside of class, lab, and quiz section, and grading part or all homework assignments, labs, and exams for the courses they support. While hours spent vary depending on the TA, the minimum average contact hours that TAs have with students is close to ten hours per week (including four hours of in-class or in-lab contact, four hours of office hours or additional review sessions, and an additional two hours of contact over email and discussion forums). Many TAs, however, interact even more often with students. While exact demographics of TAs are not available, TAs are primarily graduate students with similar demographics to the remaining graduate student population in engineering (predominantly Non-Hispanic White or Asian and male). All incoming graduate students attend a 30–45 minute orientation given by an experienced, lead TA for the department who covers logistics and expectations for TAs. For the time periods associated with this study, this orientation as well as informal one-on-one mentoring from more senior TAs and voluntary enrollment in institution-wide TA professional development training were all that was provided to TAs to prepare them for the classroom. Data regarding which TAs in this study attended voluntary additional professional development training was not available. Since the completion of this study, however, TAs now complete an institution-wide series of training and professional development modules entitled “Strategies for TAs” as well as in-person training coordinated by the lead TA with help from faculty and the institutional Center for Teaching and Learning.

### Procedures

IRB (Internal Review Board) approval (STUDY00000378) was obtained to recruit and survey undergraduate students beginning on October 26, 2016. While the exempt status under which this study was approved did not require continuing review, data collection was discontinued on June 15, 2023. Instructors were asked to offer the survey to their students within two to three weeks of the end of the term in which the course was offered. Instructors offered an incentive to students to complete the survey, with a nominal amount of extra credit being the most popular choice; extra credit has been shown to be a highly effective motivator for college students [34]. For all but one class in the pre-COVID and ERT time periods, the survey was hosted by an institution-specific survey tool (Catalyst WebQ) and students accessed and completed the survey via a link in the learning management system for the course (Canvas) within one to three weeks of the instructors publishing the survey. In the remaining course (a 2016 pre-COVID offering), students completed a paper version of the survey in class. In the post-COVID period, student responses were collected using either Catalyst WebQ (2022) or Google Forms (2023). Instructors were not provided with any survey responses but instead were provided with a list from the researchers of names and percentage of questions completed by each student so that grades could be adjusted according to the incentive offered to students. All participation was voluntary, and students were offered credit regardless of whether they granted consent for their responses to be used in the research because institutional IRB required that those students who did not provide consent not be excluded from taking the survey. Participants provided informed consent either in written form (for the paper form of the survey in the 2016 pre-COVID course) or in electronic form via a unique link to their student network ID (in all subsequent surveys). No minors participated in the survey and less than 5% of those who completed the survey did not offer consent and were eliminated from the dataset.

### Data analysis

Textual responses to the short answer question regarding TA support (hereafter referred to as the dataset) were analyzed using thematic analysis. Thematic analysis [35] is a method of qualitative data analysis which requires reading through a dataset and identifying topics, patterns, or meanings in the data that emerge repeatedly in the dataset. These patterns are then consolidated into themes that are central to the focus or goal of a study. The flexibility of thematic analysis is well suited to qualitative educational research.

In this study, thematic analysis was initially applied without relying on any underlying assumptions about what students would say. Once patterns in the data were identified, related patterns were combined into themes to describe the data. Due to the complexity of the TA support dataset, another pass at the data within certain themes was necessary to highlight distinct topics (secondary themes) within each theme that aligned with accepted practices of teaching and learning. Responses that were not classified into any of the primary themes were coded as “other” (student responded but the response was not a good fit to any of the primary themes), no response (student did not answer the question), or no suggestion (student did not think TAs needed to do anything differently). No response and no suggestion responses were combined into a single category (non-response) and reported alongside primary themes to enable consideration of the limitations imposed by non-response bias in different demographic groups. Up to two themes could be applied to each student’s response, with most student responses assigned only a single primary theme.

Once primary themes and secondary themes were identified to fully describe the dataset, the primary coder prepared a description of each theme and a randomly selected subset of 100 student responses to enable assessment of inter-rater reliability. A second researcher then coded the responses in the subset according to the theme descriptions and labels provided. Inter-rater reliability was assessed using Cohen’s kappa (κ) which measures agreement between two raters while correcting for how often the raters might agree by chance [36].

After theme assignments were finalized and tallied, chi-square tests of independence were used to evaluate potential differences in primary themes. Chi-square analyses considered three genders (male, female, other); five race/ethnicities (Black, Latino/a, Asian/White, Asian, and White), and two student statuses (domestic, international) across four different periods (all time, pre-COVID, during COVID/ERT, and post-COVID). Non-responses were also considered in the chi-square analyses under the assumption that non-responses largely reflected that students found TAs insufficiently relevant to their experience to offer suggestions for improved support.

## Results

### Pre-entry student characteristics

#### GPA.

Students in this study reported a mean GPA of 3.46 (SD = 0.32) prior to completing the course in which they completed the survey. No significant differences in GPA emerged at the p < .05 level across gender (F (1, 1575) = 0.333, **p* *= .564). However, significant differences did emerge in GPA across race/ethnicity (F (7, 1472) = 8.57, **p* *< 0.001). Subsequent post-hoc Tukey comparisons among domestic students indicated that the GPA of Asian students (M = 3.46, SD = 0.31) was significantly higher than that for Black students (M = 3.28, SD = 0.50), *p* = 0.04, Cohen’s d = 0.57 and for Latino students (M = 3.26, SD = 0.27), *p* < 0.001, Cohen’s d = 0.64. The GPA of White students (M = 3.46, SD = 0.29) was also significantly higher than that for Black students (M = 3.28, SD = 0.50), *p* = 0.04, Cohen’s d = 0.60 and for Latino students (M = 3.26, SD = 0.27), *p* < 0.001, Cohen’s d = 0.67. The GPA for other URM students (M = 3.43; SD = 0.30) was also significantly higher than that for Latino students (M = 3.26, SD = 0.27), *p* = 0.04, Cohen’s d = 0.50. Significant differences also emerged in GPA across international status. International students (M = 3.56, SD = 0.29) reported a higher GPA on average than domestic students (M = 3.44, SD = 0.32), *p* < 0.001, Cohen’s d = 0.40. All effect sizes were medium except for the comparisons of the GPAs between other URM and Latino students and between domestic and international students where the effect size was small.

#### Family income.

Family income was reported in eight brackets: $0-$10k; $10k-$20k; $20k-$40k; $40k-$60k; $60k-$80k; $80k-$100k; $100k-$150k; and greater than $150k. The largest proportion of students (22.4%) reported annual incomes associated with their family of origin in the bracket between $100k and $150k. Chi-square tests of independence for family income brackets did not find any significant differences by gender or by race/ethnicity, but differences by country of origin were significant [*χ*^2^ (6, 1550) = 50.8, **p* *< 0.001, Cramer’s V = 0.19 (medium effect size)]. Lower family of origin incomes were more prevalent among international than domestic students, but these differences may not correspond to lower socioeconomic status due to differences in the cost of living between the U.S. and other countries.

#### Father’s education.

Since mother’s education and father’s education were significantly correlated, only father’s education results are reported. No significant differences by gender or race/ethnicity emerged from the data but country of origin [*χ*^2^ (6, 1631) = 34.6, **p* *< 0.001, Cramer’s V = 0.15 (medium effect size)] differences were significant with fewer international students originating from families of lower educational levels than domestic students.

### Themes of TA support

A first pass at the data indicated that 9.6% of students offered responses that were coded into multiple themes. Of all themes assigned to student responses, students most often expressed preferences for TA support associated with *interactions* (42.9%), followed by *preparation* (37.6%), and *care and hospitality* (7.61%). The inter-rater reliability of these three primary themes was assessed by measuring Cohen’s kappa of a dataset coded by two researchers trained in engineering education research. Results of the analysis are shown in Table 3. For this dataset, Cohen’s kappa was determined to be 0.815 which is considered substantial [36] even after considering that the presence of more than two themes in this study introduces a positive bias (i.e., an inflated value of Cohen’s kappa).

**Table 3:** Inter-rater reliability.

	Rater #2			
Rater #1	Practice	Preparation	Hospitality	Percentage Agreement
Practice	46	1	0	97.9
Preparation	9	39	0	81.3
Hospitality	1	0	8	88.9

Observed agreement between raters ($p_{o}$): 0.894.

Probability of chance agreement between raters ($p_{e}$): 0.428.

Cohen’s kappa: 0.815.

**Fig 2:**
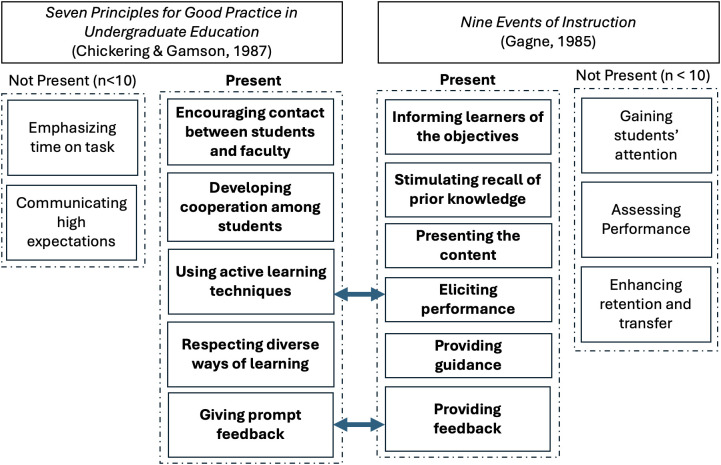
Secondary themes associated with interactions and preparation.

Within themes of *interactions* and *preparation*, several secondary themes emerged in the data. We situated these secondary themes within two frameworks of teaching and learning: the seven principles of good teaching in undergraduate education outlined by Chickering and Gamson [37] and the nine essential instructional events outlined by Gagné [38]. Five of the seven practices of good teaching (two of which overlap with Gagné’s nine instructional events) and four additional instructional events were expressed by at least ten students in the study population (Fig 2).

The secondary themes corresponding to five of the seven best practices for teaching set forth by Chickering and Gamson [37] and two of Gagné’s nine instructional events [38] were:

- *Encouraging contact between students and faculty/instructors (Contact):* Frequent contact between teacher and student is “…the most important factor in student motivation and involvement.” (37, p. 3) and in this study, included office hours, interaction in discussion forums, emails, and supplemental review sessions as well as informal conversations between TAs and students.
- *Developing cooperation among students (Collaboration):* As part of good teaching practice, instructors can facilitate both formal and informal teams of students to leverage the value of collaboration in the social aspect of student learning.
- *Using active learning/Eliciting performance*: Team projects, peer critiques, challenging discussions, and structured, student-centered problem-solving exercises all serve to move learning away from being a spectator sport to a process in which students are actively engaged.
- *Giving prompt feedback/Providing feedback:* Frequent feedback from teachers, whether through formal, graded assignments or more informally through prompt responses to questions posed by students, is a critical part of academic performance and learning.
- *Respecting diverse ways of learning:* Bringing diverse ways to solve problems, complete labs, or excel in classes respects differences among students in their individual talents and ways of learning.

Within the primary theme of *preparation*, four secondary themes (corresponding to four of Gagné’s nine instructional events) also emerged:

- *Informing learners of the objectives:* Communication of learning objectives, whether through explicit learning outcomes or through summaries of what will be or what has been accomplished, helps to focus students’ attention and improve motivation.
- *Stimulating recall of prior knowledge*: Reviewing relevant concepts of the main faculty lecture or referring to knowledge associated with previous courses enables students to leverage what they already know.
- *Presenting the content*: Ensuring clear, organized delivery, using appropriate teaching strategies, and providing supplemental learning resources establishes a necessary foundation for learning. This secondary theme was considered part of preparation since it involves considerable planning and for young teachers as TAs often are, considerable practice to ensure clear, audible, and articulate communication.
- *Providing guidance*: Examples, lab demonstrations, and other similar practices help students grasp and reinforce content.

The last primary theme that emerged from the data is that of care and *hospitality* where “… teaching with hospitality refers to the ability of the professor to provide a nurturing, conducive learning environment” [39, p. 10.376.8] and includes “…listening with respect, receptiveness to other opinions, and requiring the same level of interaction and courtesy from all students.” [39, p. 10.376.8]. Students reflecting on a need for hospitality in the classroom referred to seeking TAs who were understanding, caring, flexible, enthusiastic, and interested (among other descriptors) in their teaching practice. Although student responses that emphasized hospitality were distinct and merited a separate, primary theme in interpreting our data, no secondary patterns or themes associated with hospitality emerged.

Sample sizes among secondary themes were substantially smaller than those associated with primary themes, so neither inter-rater reliability nor chi-square tests were performed on these secondary themes. Insight into the nature and meaning of these secondary themes, however, was available through qualitative analysis of student responses, described in more detail next.

### Interactions

In the overall study population, 789 (42.9%) of primary themes expressed in student responses called for TAs to make it a priority to facilitate high-quality interactions either between TA and student or among students (Table 4).

**Table 4:** Students who desire interactions with TAs.

Demographic	All Time Periods		Pre-COVID		ERT		Post-COVID	
	*N*	%*	*N*	%*	*N*	%*	*N*	%*
All Responses	789	42.9	182	30.7	431	51.6	176	42.7
*Gender (among all students/participants)*								
Male	573	42.8	130	29.6	310	52.1	133	43.9
Female	205	43.0	50	34.0	116	50.7	39	39.4
Other	7	50.0	2	100	3	42.9	2	50.0
*Race/Ethnicity (among domestic students/participants)*								
Black	12	35.3	3	30.0	7	41.2	2	28.6
Latino	24	34.8	4	5.00	13	41.9	7	35.0
Other URM	59	47.6	15	35.7	31	62.0	12	46.2
Asian	241	44.6	47	31.3	127	52.9	67	44.7
White	291	43.8	83	32.4	155	53.5	53	44.9
Asian/White	40	45.5	9	33.3	20	44.4	11	68.8
*Country of Origin (among all students/participants)*								
Domestic	679	43.9	162	31.8	363	52.7	154	44.0
International	105	37.5	20	24.1	65	46.4	20	35.1

*Percentage of assigned primary themes (codes) within a demographic and time period.

Such preferences were most frequently cited during the COVID-19 pandemic with 431 of assigned, primary themes (51.6%) reflecting an expectation that TAs focus on facilitating high-quality *interactions*. Across demographics, *interactions* appeared to be top priority for fewer Black (35.3%) and Latino (34.8%) students than for Asian (44.6%), White (43.8%), and Asian/White (45.5%) students. Domestic students also favored *interactions* in their responses (43.9%) more than international students (37.5%). Responses across gender were relatively balanced with 42.8% of male responses and 43.0% of female responses calling for improvements in interactions facilitated by TAs.

Of the five secondary themes identified within interactions, students most frequently called for more *contact* with TAs (72.2% of the 789 responses coded as *interactions*). Many students referred to office hours as an effective way for TAs to maintain contact with students:

Providing a good amount of time for office hours especially during busy weeks… Since the class has labs to do outside of the class, students would often work on it during the TA office hour times for extra help when needed. But when the lab is long, TAs are often overloaded with questions. Having extended office hours would be nice *(domestic Asian female, electrical and computer engineering major)*

Other students prioritized contact with TAs by calling for increased responsiveness:

Provide quick and active support when needed, like regularly checking and responding to emails (*domestic other URM male, electrical and computer engineering major*)

Still other students were open to a range of ways to maintain contact with TAs:

An action that TAs can do to support me in my classes is to provide assistance through various mediums of communications *(domestic Asian male, electrical and computer engineering major)*

Be available to help as much as possible, whether through email or meeting *(domestic White male, electrical and computer engineering major)*

Communicating with each student individually so that they know exactly where their understanding is. Reaching out and being available for each student to ask questions… *(domestic White male, aeronautical engineering major)*

When referring to *interactions*, students also called for better or more frequent *feedback* from TAs (9.13%). These students desired faster or more direct formative feedback on graded assignments:

Providing comprehensive feedback on assignments *(domestic Asian male, electrical and computer engineering major)*

Depends on class but faster grading for assignments so that we can improve our work as we go through the quarter *(domestic Asian male, electrical and computer engineering major)*

Preliminary checks on assignments before being graded *(domestic Asian male, electrical and computer engineering major)*

Provide feedback about how you can improve if you do poorly *(domestic Asian male, electrical and computer engineering major)*

While *feedback* and *contact* were common secondary themes among student responses within the *interactions* theme, a meaningful number of students also desired that their TAs provide more active learning experiences (13.4%):

Let us do things instead of just talking through examples. That’s why we have lecture so it’s much better to learn through practicing and asking questions when we have the TAs’ time *(domestic White female, mechanical engineering major)*

Some students also sought opportunities for increased *collaboration* with peers (2.75%):

Encourage students to introduce themselves to each other (e.g., in labs or when asking students to participate in class) *(domestic male, electrical and computer engineering major)*

and more *diverse* teaching methods and problem-solving approaches (2.38%) in interactions with students:

Offer alternative explanations of concepts rather than repeating ones that students don’t understand *(domestic White male, physics major)*

Offer explanations in a different manner than the professor. Make alternate explanations available *(domestic White male, industrial engineering major)*

Be ready to explain a solution/problem more than one way *(domestic White male, civil engineering major)*

While *interactions* dominated student preferences for TA support, responses that called for improved *preparation* were also prevalent in the study population, discussed next.

### Preparation

The second most popular theme among student responses regarding TA support was that of *preparation*. 37.6% of student responses called for some form of improvement in what TAs should do to prepare for providing high-quality learning experiences to students (Table 5). TA preparation was most popular among Latino students (43.5%) as well as female (40.7%) and Black (41.2%) students and was viewed as a priority to more students prior to the COVID-19 pandemic (47.4%) than during (31.4%) or after (36.2%) the pandemic.

**Table 5:** Students who desire better preparation from TAs.

Demographic	All Time Periods		Pre-COVID		ERT		Post-COVID	
	*N*	%*	*N*	%*	*N*	%*	*N*	%*
All Responses	692	37.6	281	47.4	262	31.4	149	36.2
*Gender (among all students/participants)*								
Male	493	36.9	210	47.7%	175	29.4	108	35.6
Female	194	40.7	71	48.3%	85	37.1	38	38.4
Other	3	21.4	0	0.00%	2	28.6	1	25.0
*Race/Ethnicity (among domestic students/participants)*								
Black	14	41.2	4	40.0	7	41.2	3	42.9
Latino	30	43.5	10	55.6	13	41.9	7	35.0
Other URM	41	33.1	18	42.9	13	26.0	6	23.1
Asian	208	38.5	75	50.0	78	32.5	55	36.7
White	250	37.7	119	46.5	86	29.7	45	38.1
Asian/White	35	39.8	12	44.4	18	40.0	5	31.3
*Country of Origin (among all students/participants)*								
Domestic	586	37.9	239	47.0	219	31.8	128	36.6
International	102	36.4	42	50.6	41	29.3	19	33.3

*Percentage of assigned primary themes (codes) within a demographic and time period.

Among those students who prioritized *preparation*, a majority wanted TAs to improve the *presentation of content* for their learning (50.5% of the 692 responses coded as *preparation*). Some of these students called for more professional, clear, and organized delivery from TAs both in planning what they say and how they say it:

In this class specifically, the TAs move way to fast in their teaching and are super unclear. They do not cater well to people who have never worked with a program like this. They skipped over the basics and assumed I knew what I was doing, which set me up for failure because it took weeks before I was able to do basic things in the program I should have learned in the very beginning *(domestic White female, mechanical engineering major)*

Be able to explain concepts explained in lecture and break them down to more understandable terms *(domestic Latino female, electrical and computer engineering major)*

More specifically, some students called for TAs to be better prepared to expand on primary course content:

Discuss more lecture materials because in lecture things are covered so fast and I cannot fully follow that *(international Asian female, electrical and computer engineering major)*

One action our TAs can take to support us in our classes is to clarify any misunderstandings that the professor made in lecture … *(domestic Asian male, electrical and computer engineering major)*

Other students explicitly called for better content presentation with respect to labs:

I would really like specifics on lab content because some of us have never used a breadboard, transistors, etc. *(domestic White female, electrical and computer engineering major)*

TA should explain more about the lab instead of just letting us do the labs by ourselves *(domestic Asian female, electrical and computer engineering major)*

A substantial number of students (41.7% of the 692 responses coded as *preparation*) also called for TAs to provide better *guidance*, both in terms of examples:

Thoroughly work through difficult problems with students while providing commentary on their thought process for solving the problem *(domestic Asian male, mechanical engineering major)*

Providing “cheat sheets” or extra practice problems is very useful. We students need more guidance on concepts (*domestic White male, electrical and computer engineering major*)

and using good questioning strategies:

Answer questions more directly. Too often I’ve had TA’s who give totally indirect answers to homework questions, and it doesn’t serve any purpose except to frustrate students. I want to be clear, that I’m not asking them to just give up answers, but I feel that too many TA’s avoid giving actual assistance by dodging questions with more questions *(domestic White male, mechanical engineering major)*

As well as clarifying rubrics for assessment and grading:

Clearly present the expectations and grading rubric of the professor (*domestic White male, electrical and computer engineering*)

Provide specific requirements and rubrics on assignments so grading is not subjective (*domestic White male, civil engineering major*)

While over 90% of students who called for better *preparation* from TAs expressed expectations that aligned with secondary themes of *presenting content* and providing *guidance*, a few explicitly called for TAs to contextualize what they were learning in terms of course *learning objectives* (2.78%) and emphasize *recall of prior knowledge* in lecture or in previous courses (2.20%). Fewer than ten students expected TAs to gain their attention in class, develop or adjust grading and assessment practices, or engage students in learning transfer.

While both *preparation* and *interactions* and their secondary themes were important to a substantial number of students with regard to how TAs should support their engineering education, the degree of care and *hospitality* TAs expressed in how they handled their interactions with and presentation to students was also important. This theme is discussed next.

### Hospitality

Among all student responses to TA support preferences, 7.61% called for some form of improvement in TA *hospitality* (Table 6). Underrepresented minority students (Latino (11.3%), other gender (14.3%), and other URM (11.5%)) emphasized hospitality most frequently while response rates for other demographics (White, Asian, Asian/White, domestic, international, male, female) were less than 8.00%. Further, among the three time periods studied, calls for hospitality were least common during the COVID-19 pandemic/ERT (4.43%) and above 10.0% prior to and after the pandemic.

**Table 6:** Students who desire hospitality from TAs.

Demographic	All Time Periods		Pre-COVID		ERT		Post-COVID	
	*N*	%*	*n*	%*	*N*	%*	*n*	%*
All Responses	140	7.61	60	10.1	37	4.43	43	10.4
*Gender (among all students/participants)*								
Male	98	7.32	45	10.2	26	4.37	27	8.91
Female	38	7.97	15	10.2	9	3.93	14	14.1
Other	2	14.3	0	0.00	1	14.3	1	25.0
*Race/Ethnicity (among domestic students/participants)*								
Black	2	5.60	1	10.0	1	5.88	0	0.00
Latino	8	11.3	2	11.1	2	6.45	4	20.0
Other URM	14	11.5	5	11.9	3	6.00	7	26.9
Asian	38	7.04	16	10.7	11	4.58	11	7.33
White	48	7.17	23	8.98	13	4.48	12	10.2
Asian/White	7	7.87	4	14.8	3	6.67	0	0.00
*Country of Origin (among all students/participants)*								
Domestic	122	7.88	53	10.4	33	4.80	36	10.3
International	18	6.43	7	8.43	4	2.86	7	12.3

*Percentage of assigned primary themes (codes) within a demographic and time period.

Some expectations for hospitality provided insight into why students wanted a TA to act in certain ways:

Be more friendly and vocal. Be more willing to help and enthusiastic about what they do. This will motivate students to work harder in the class and enjoy the course *(international Asian male, civil engineering major)*

Be more approachable. This can be done by making it very clear through body language, and emotion, that they’re interested in our success and are willing to help us to get be successful. This is hard if a TA isn’t interested, but if they are, being as genuine as possible with students helps us (or me at least) feel like they’re approachable, which makes me want to succeed more *(domestic male, electrical and computer engineering major)*

In contrast, other students simply asked for a range of positive personality characteristics:

Be welcoming and encouraging in the engagement of learning *(domestic Asian male, electrical and computer engineering major)*

Be kind and relatable *(domestic White male, electrical and computer engineering major)*

Really be open and expressive about their want to help *(domestic Asian male, mechanical engineering major)*

Being understanding is exceptionally important, in my honest opinion *(international Asian male, electrical and computer engineering major)*

And still other students hinted at negative experiences with TAs that they would like to see changed:

They do a good job so far, but many don’t have enough patience. They often just want to give us the answer instead of finding the root of our misunderstanding (*domestic male, civil engineering major)*

Be understanding and helpful. It is so annoying when they are like “well I can’t just give you the answer, and you need to solve this on your own”. I came to office hours because I can’t solve it on my own *(domestic Asian male, mechanical engineering major)*

Not be condescending when the students don’t understand the material well *(domestic Asian male, civil engineering major)*

### Non-responses

A non-trivial proportion of students (8.32% overall) chose not to answer the question regarding expectations for TA support or had no suggestions (Table 7). The lowest non-response rates were among females (5.03%), Asian/White (4.55%) and other URM students (3.23%) while the highest non-response rates occurred among International (16.1%) and Black (17.7%) students.

**Table 7:** Students who did not respond to the TA support question.

Demographic	All Time Periods		Pre-COVID		ERT		Post-COVID	
	*n*	%*	*n*	%*	*N*	%*	*n*	%*
All Responses	153	8.32	32	5.40	82	9.82	39	9.47
*Gender (among all students/participants)*								
Male	125	9.34	27	6.14	67	11.3	31	10.2
Female	24	5.03	4	2.72	13	5.68	7	7.07
Other	1	7.14	0	0.00	1	14.3	0	0.00
*Race/Ethnicity (among domestic students/participants)*								
Black	6	17.7	2	20.0	2	11.8	2	28.6
Latino	5	7.25	1	5.56	2	6.45	2	10.0
Other URM	4	3.23	1	2.38	2	4.00	1	3.85
Asian	39	7.22	7	4.67	16	6.67	16	10.7
White	42	6.33	10	3.91	26	8.93	6	5.08
Asian/White	4	4.55	1	3.70	3	6.67	0	0.00
*Country of Origin (among all students/participants)*								
Domestic	104	6.78	23	4.52	53	7.69	29	8.29
International	45	16.1	8	9.64	28	20.0	9	15.8

*Percentage of assigned primary themes (codes) within a demographic and time period.

### Demographic differences

The statistical significance of differences in response patterns across demographics for TA support were evaluated using chi-square tests of independence. Results shown in Table 8 indicate whether the null hypothesis could be rejected. For example, chi-square tests across gender evaluated whether the hypothesis that male and female students responded in similar proportions could be rejected. Chi-square tests indicated that this null hypothesis could be rejected when considering the entire student sample and the ERT sample, but when considering the pre-COVID and post-COVID sample, the null hypothesis could not be rejected. Non-binary students were not included in the chi-square tests by gender because including this group appeared to mask gender differences in male and female students even when using simulated *p*-values to compensate for small sample sizes.

**Table 8:** Results of pearson’s chi-square tests of independence.

			*Standard Chi-Square*			With simulated p-value*
Time Period	Student Demographic	*N*	*χ* ^2^	df	*p*	*P*
All	**Gender (Male, Female)**	**1750**	**9.50**	**3**	**0.023**	**0.025**
	**Race/Ethnicity**	**1403**	**28.48**	**12**	**0.005**	**0.005**
	**Country of Origin**	**1762**	**27.94**	**3**	**<0.001**	**<0.001**
	**Time Period**	**1774**	**88.83**	**6**	**<0.001**	**<0.001**
Pre-COVID	Gender (Male, Female)	552	3.102	3	0.376	0.381
	Race/Ethnicity	433	7.156	12	0.847	0.853
	Country of Origin	554	5.468	3	0.141	0.128
ERT	**Gender (Male, Female)**	**801**	**8.554**	**3**	**0.036**	**0.037**
	Race/Ethnicity (Domestic)	603	6.312	12	0.900	0.901
	**Country of Origin**	**806**	**19.88**	**3**	**<0.001**	**<0.001**
Post-COVID	Gender (Male,Female)	397	3.292	3	0.349	0.349
	Race/Ethnicity (Domestic)	308	15.88	12	0.197	0.187
	Country of Origin	402	4.241	3	0.237	0.250

*Degrees of freedom (df) are not relevant in simulated Chi-square tests.

**Fig 3:**
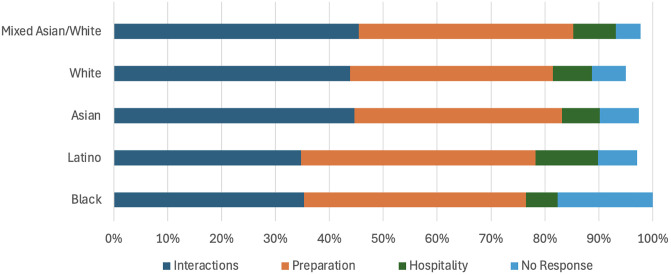
Race/Ethnicity differences in student preferences for TA support (all time periods). Totals do not add up to 100% because some responses were sufficiently ambiguous that they could not be accurately coded according to the four primary themes.

The results of other chi-square tests indicated that there were significant differences by student race/ethnicity when considering all time periods (*χ*^2^ (12, *N* = 1,403) = 28.48, *p* = 0.005). Particularly noteworthy, a high proportion of Black students did not offer any suggestions for TA support (i.e., no response). Further, Latino and Black students disproportionately favored *preparation* and Latino students disproportionately favored *hospitality* in their responses (Fig 3). Other URM students were not included in the chi-square analysis by race, because they represent a heterogenous group of races (i.e., it cannot be assumed that all students within the Other URM group are bringing similar racial experiences to the table).

**Fig 4:**
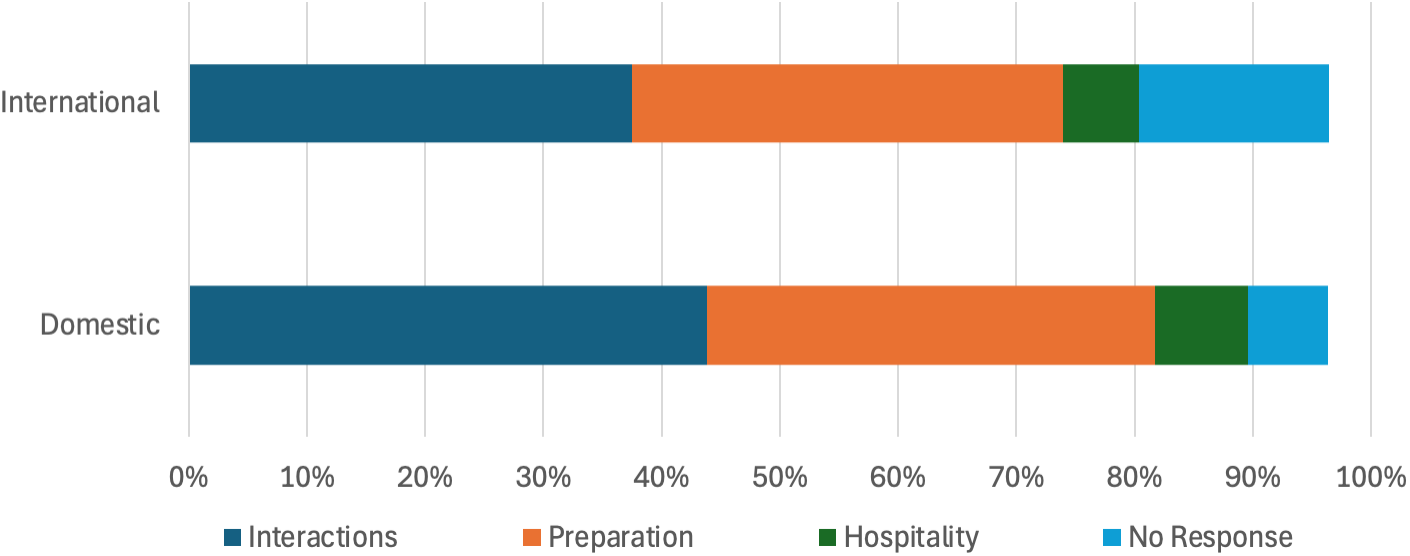
Country of origin differences in student preferences for TA support (all time periods). Totals do not add up to 100% because some responses were sufficiently ambiguous that they could not be accurately coded according to the four primary themes.

When considering students’ country of origin, significant differences also emerged for all time periods (*χ*^2^ (3, *N* = 1762) = 27.94, *p* < 0.001). A disproportionately high number of international students did not offer an answer to the TA support question and domestic students called for improvements in *hospitality* and *interactions* in higher numbers than their international peers (Fig 4).

**Fig 5:**
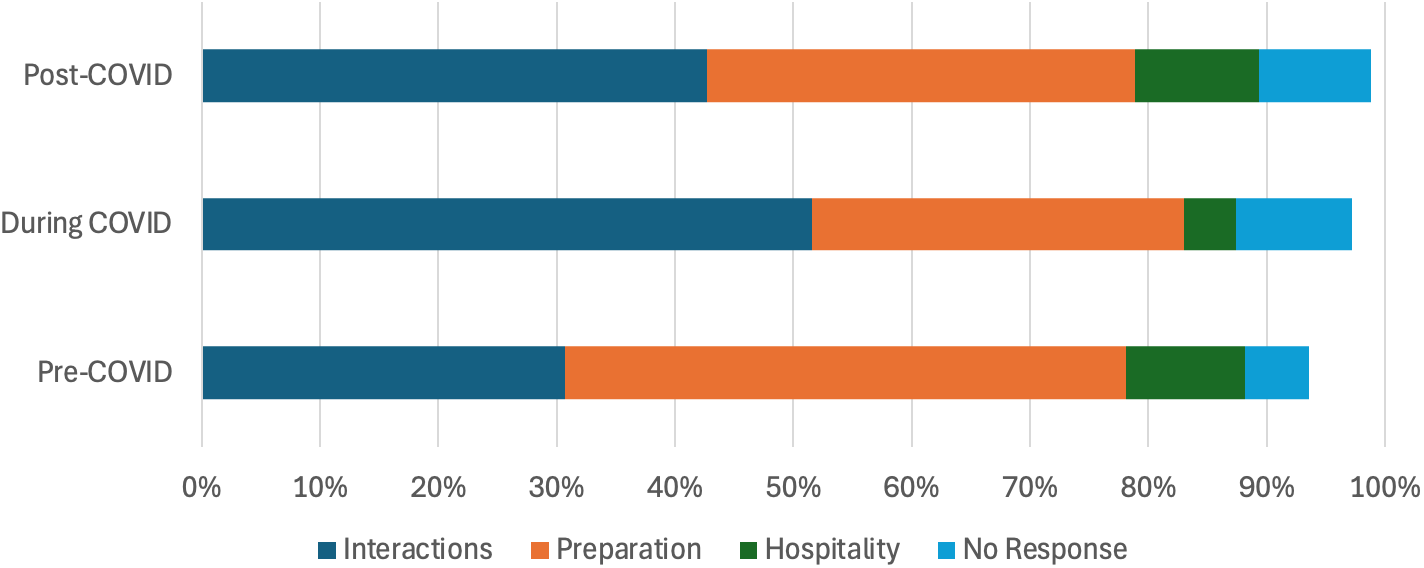
Time period differences in student preferences for TA support. Totals do not add up to 100% because some responses were sufficiently ambiguous that they could not be accurately coded according to the four primary themes.

Student expectations for TA support also varied across different time periods (*χ*^2^ (6, *N* = 1774) = 88.83, *p* < 0.001). Students disproportionately favored *interactions* during the pandemic; *hospitality* before and after the pandemic; and *preparation* prior to the pandemic (Fig 5).

## Discussion and implications

The results of this study indicate that what students expect from TAs is not fixed but varies during different time periods of instruction (pre-COVID vs. post-COVID), in different classroom conditions (traditional classroom format vs. ERT format), and with the diversity of thought and lived experiences that students bring into the classroom.

### RQ1: What do undergraduates expect from TAs?

A significant majority of students (80.5%) prioritized *interactions* and *preparation* in their expressed expectations for TAs. Considering the entire study population, the most frequent requests from students (expressed as a percentage of the total assigned primary themes/codes) were for increased *contact* with TAs (31.2%), improved content presentation (19.0%), better guidance (15.7%), and greater hospitality (7.61%). Engineering students want TAs to be available, to spend time helping them to solve problems, to troubleshoot, to provide clear and organized content to them, and express care through friendly, respectful and kind behaviors. A desire for frequent and meaningful communication with TAs is consistent with previous studies that showed TAs are perceived as interactive and relatable partners in the personalization of teaching [19]. These results are also consistent with previous studies of pre-COVID and pandemic era classrooms that showed students most frequently call for increased *interactions* with TAs in their preferences for TA support [40]. However, while frequent contact with TAs was critically important to many students, elements of teacher *preparation* (content presentation and guidance) were also frequently mentioned in student responses. These results are consistent with previous studies that highlighted the importance of high-quality in-class delivery by TAs [40], but this study has provided additional, more granular information regarding what is particularly important to students in the classroom. Students specifically called for TAs to align with and supplement the teaching of the primary instructor/professor by being well versed in and reviewing lecture material as well as by guiding students through examples that empower them to learn and perform well on homework, tests, labs, and other assignments. These student expectations for high-quality, in-class teaching from TAs are at odds with perceptions from students that TAs are often “hesitant, nervous, uncertain, and unsure how to begin teaching.” [19, p. 196]. Thus, this study has reinforced the need for TAs to have ample opportunities to practice and develop their presentation and delivery skills prior to beginning in-classroom teaching.

The results of this study also highlight the gap between student and TA expectations for teaching and support. While research shows that TAs believe that content knowledge is the sole key to being an effective teacher [13], students have a much different idea of what TAs should bring to the table. In our study, only 19.0% of students focused on content in their responses to TA support. This result is consistent with a previous study of seven laboratory and lecture courses in environmental and water resources engineering where students were asked to rank what makes an effective TA from 17 categories of intellectual excitement and interpersonal rapport developed by the American Society of Civil Engineers Body of Knowledge [41]. 21.3% of students ranked fair grading practices as their first choice for what makes for an effective TA followed by explaining difficult concepts well (14.9%), coming to the classroom or laboratory prepared (13.3%), communicating clearly (12.9%), being available outside of class (7.2%), and treating all students with respect (6.0%) [41]. Being an expert in the content area was ranked as the first choice for effective TA teaching by only 6.0% of students in this study, reinforcing the notion that what TAs think they should be doing may be very different from what students expect them to do.

### RQ2: Does a student’s race, gender, or international status affect their expectations of TAs?

Several themes emerged from the student demographic differences that emerged in our study. Among them is the emphasis on *preparation* that underrepresented minorities (URMs) place on TA support. In particular, a higher proportion of Latino and Black students than other students called for improved teacher *preparation* from their TAs (Table 5). Latino students also called for *hospitality* from TAs at higher frequencies than their peers (Table 6). There are several reasons why teacher *preparation* and *hospitality* may be more important to many underrepresented minorities (and by extension why teaching practice may be less important). One possibility is that the underlying lack of belonging that these underrepresented groups feel in engineering majors [42,43] causes them to value hospitality from their instructors (including TAs) more than their majority peers (to offset a sense of not being accepted or included). Lack of belonging or feelings of isolation may also lead to a reluctance to interact with TAs and benefit from the interactive support that is emphasized in teaching practice. Instead, these students may turn to relying more on what the TA delivers in terms of presentation of content and formal guidance. Empirical evidence has supported this possibility by demonstrating that underrepresented students are indeed less likely to interact with faculty and instructors [44,45].

For Latino students, other factors may also contribute to a disproportionate reliance on teacher preparation. For example, Latino students report working more outside of school to support their education and family [46] which would limit their time to take advantage of additional office hours, review sessions, and other out-of-class contact that TAs may offer as part of their teaching practice. The fact that there were no significant differences in family income in our study population by race/ethnicity, however, suggests that outside work may be less of a contributing factor in this study. Furthermore, in this study population, the GPA of both Asian and White students was significantly higher than that of Latino students, which suggests at the very least that Latino students may need different forms of TA support than their Asian and White peers. This may do in part to the lack of access that Latino students have to non-novice teachers in high school [47] or it may be due to differences in expectations for college teaching that emerge from a broader range of pre-entry, lived experiences.

With regard to gender, it is noteworthy that in contrast to previous studies, this study suggests gender differences in student expectations for instructional support. When studying faculty support, Belcheir [48] found no gender differences when considering students’ expectations for instructor behaviors related to feedback, examples, and lesson preparation. Belcheir’s study did not consider contact (interactions with instructors), but other studies that specifically focused on faculty-student interactions have not found gender differences either [45]. In contrast in this study, 40.7% of females and only 36.9% of males emphasized *preparation* (Table 5), indicating that female students may find this more important than male students do. These differences may be a result of how the questions were posed to students (open-ended in this study vs. closed form in other studies), changes that have occurred since Belchier’s study was published (1998), or unique preferences for instructional support in engineering compared to previous studies that cover a broader range of disciplines. Also noteworthy among gender differences, 9.34% of male students chose not to respond to the TA support question at all, compared to 5.03% of female students (Table 7). This result may imply that more male students are generally satisfied with TA support (and see no substantial room for improvement), or more likely, it suggests that more males than females do not rely on TA support as a critical element of their learning. Regardless of the reason, the gender differences in this study compared to the lack thereof demonstrated in previous studies underscores the need for further study into how male and female students view the role of TAs in specific disciplines.

In addition to the demographic differences across gender and race/ethnicity that emerged from this study, our data has also added to existing evidence that international students have a different set of expectations and experiences in engineering than domestic students do. Of particular interest is that a high percentage of international students did not respond to the question regarding TA support (16.1%, Table 7). Like underrepresented domestic students, this may also stem from a lower sense of belonging but is likely to be compounded by experiences of bias and discrimination, loneliness, and homesickness [49] and other barriers to integration into the campus experience that are commonly reported by international students [50]. Whether or not language barriers come into play, international students may be reluctant to interact with TAs and other instructors and may be unsure of what to expect from TAs, hence leading to a poor response rate when asked about TA support. Since more international than domestic students come from higher income families with more advanced degrees and report a higher GPA on average than their domestic peers, it is also possible that many of these international students do not rely on TA support as much as other students. These students may have figured out how to do engineering classes “on their own”, thereby relying on TAs far less for their academic success.

Other demographic groups in this study also exhibited high non-response rates. Black and Latino students did not respond to the TA support question more frequently than White students, a result that has been replicated in surveys of the general population in the United States [51] but females responded at higher rates than males, which is opposite to trends in the general population [51]. Whether the lack of a response reflects students’ perception that TA support is irrelevant, students’ lack of motivation to answer open-ended questions, or some other reason merits further study to understand and reduce non-response bias in the interpretation of the results.

It is important to consider that doing everything that students ask for is not likely to work for TAs in terms of available time and workload, thus making it critically important to identify what is most important to students so that TAs can focus their efforts into areas of highest impact. Thus, professional development for TAs should include a static training component on effective pedagogy as well as a more dynamic component that guides TAs towards diagnosing (via surveying or other means) what students want at the start of their courses and tailoring their teaching efforts accordingly.

Additionally, while this study explores student expectations on the premise that meeting them at a meaningful level is important, we do not advocate that TAs base their teaching strategy solely on what students want. Certainly, meeting the expectations of students is likely to increase student satisfaction which positively influences academic outcomes, but TAs (and other instructors) should remain aware of the elements of high-quality teaching and instruction events that are absent from student expectations. These absent elements are represented within the two teaching and learning frameworks that were chosen to ground the findings in this study. Among the seven principles of good teaching practice [37], students only spoke to five principles; emphasizing time on task and communicating high expectations were notably absent from their responses. Emphasizing time on task is reflected in such behaviors as underscoring deadlines, providing reminders, breaking assignments into smaller, more manageable pieces, and providing frequent low stakes assignments. Communicating high expectations can be done via detailing expectations in the course syllabus, giving students detailed rubrics describing what is emphasized in grading and how, and similar behaviors. Students also did not recognize all the instructional events necessary for their learning. Three of Gagné’s nine elements of instruction [38] were missing from the students’ responses: gaining attention, assessing performance, and enhancing retention and transfer. Gaining students’ attention can be achieved through implementing ice breakers, sharing relevant items from current news and events, questioning students appropriately, designing in-class polls, and similar methods, yet implicit in student responses in this study seems to be a sense that attention is the student’s responsibility, at least when it comes to their experiences with TAs. Similarly, students do not seem to expect that assessment is the responsibility of TAs despite the fact that TAs are heavily involved in grading. This suggests that students believe assessment is largely up to the primary instructor/professor and that TAs have little say over how their learning will be evaluated. Finally, all but a small minority of students did not see it as a high priority for TAs to be agents for personalizing their learning to support the retention of what they have learned and the transfer of that learning to their future jobs and other domains. The lack of these critical elements in our dataset is concerning. Their absence may be due in part to the fact that lecture continues to predominate in engineering and other STEM classes [52] thereby constraining student expectations to a narrow set of teaching practices seen in their prior undergraduate experiences. Or the deficit may be emerging from students holding reduced expectations for TAs vs. the primary instructor/faculty for any number of reasons including sympathy for the TA’s lack of experience and empathy for their student status. Regardless of the reason(s), however, this study has offered additional evidence that training and professional development for TAs has room for improvement, particularly in how to expose inexperienced TAs to a full range of high-quality teaching practices. Further, strategically integrating these elements into teaching while attending to student expectations at a level that maintains their satisfaction is complicated and realistically, relies on high-quality mentoring from supervising faculty who have been trained in and have experience with these instructional practices.

Finally, this study also found that despite no differences in pre-entry attributes (family income, father’s education) across domestic (non-international) gender and race/ethnicity, differences in expectations for TA support emerged across certain demographics. Conceptually, this suggests that other pre-entry attributes and lived experiences may have a more significant influence on how different engineering students view TAs and what they expect from them. At the very least, however, this study has offered additional evidence that there is no “one size fits all” teaching strategy and that TAs, like faculty, must take into consideration different needs of diverse students in their teaching.

### Summary

This study has indicated that TAs are expected to be a strong ally and peer to students, while also demonstrating domain expertise and high-quality delivery. TAs are already juggling a myriad of research and student responsibilities along with teaching duties. The broad range of expectations that students place on TAs informed by the results of our analysis may be an unmanageable burden on those TAs, potentially leading to higher burnout and withdrawal from teaching. However, this study also offers insights into the specific behaviors that make a difference to more students than others. Frequent contact with and availability for students, providing fast feedback regarding student work, providing polished presentation of content and accompanying guidance on how to apply that content were all mentioned frequently by students in their responses to the TA support question posed in this study. However, not all demographics have the same expectations which may in part be due to what they bring to the table. For example, GPA (as a measure of academic performance) seems to be important in influencing what students expect from TAs. Several underrepresented groups in this study reported lower GPAs than majority (White) and overrepresented (Asian) populations and also expressed that hospitality and improved preparation (through content delivery and subsequent guidance) were particularly important to them. Thus, investing more time into teacher preparation and creating a welcoming and inclusive environment (through hospitality) has the potential to improve equity between these students and majority groups.

## Limitations

All participants in this study were from a single, large research institution in the U.S. and most were majoring in engineering. Thus, the results may not be generalizable to other institutions or majors/disciplines whose practices differ significantly from those in engineering. In particular, half of the engineering classes in this study were relatively small (less than 100 students) which may have biased students’ responses about particular themes such as hospitality. Nevertheless, the fact that nearly all student responses in this study could be classified within two accepted and generalized frameworks for teaching and learning in higher education (Chickering and Gamson [37]; Gagné [38]) suggests that the primary and secondary themes used in this study could be readily applied to investigating students’ expectations of TA and other instructional support in a broad range of majors.

Another potential limitation to this study is that the racial composition of the student population was skewed compared to overall U.S. engineering enrollment. Asian American students were substantially overrepresented (34.6%, vs. 16.1% nationally) and Black students (2.29%, vs. 5.40% nationally) and Latino/a students (4.57%, vs. 15.8% nationally) were underrepresented [1]. Despite small sample sizes for underrepresented groups, race/ethnicity differences in what students expect and prefer from TAs still emerged in our study population, suggesting that race and ethnicity should continue to be an important consideration in further research studies of this type, in TA professional development efforts, and in the development of inclusive classroom strategies. Another limitation in the demographic and student characteristic data related to the way the educational background of the students’ caregivers was phrased. Using “father’s education” as a basis for assessing educational background of the family of origin introduced some bias into the data because it is likely that some students in this study did not have heterosexual families or were raised in single parent (mother) or other non-traditional homes.

The results of this study may also be limited by the thematic analysis of text-based data (i.e., short answer questions) instead of analysis of numerical data using more objective, bias-free methods. In thematic analysis, the process of coding (identifying patterns) and assigning themes to data is highly subjective and conducive to multiple approaches to interpreting the data; as a result, it is highly vulnerable to researcher bias. While this bias was shown to be less of a concern as indicated by strong interrater reliability (κ = 0.815), it is impossible to remove all bias from qualitative analysis performed by human researchers. The richness and depth of the data collected from short answer vs. close-ended questions as well as the large sample size associated with this study, however, is likely to have offset potential inaccuracies introduced by researcher bias. Non-responses are also a potential source of bias, particularly for international students (17.7% non-response rate) and Black students (16.1% non-response rate) in this study. These non-response rates are similar to the non-response rates exhibited in the general population for short sentence and multiple sentence responses [51] and well above the 70% response rate threshold used for the National Center for Education Statistics for conducting a non-response analysis [53]. Nevertheless, understanding why some students, particularly within certain demographics, do not respond to open-ended questions about instructional support is an important topic for future research.

An additional limitation of this study is that the question posed to students prompted them to provide a single expectation of TAs that was most important to them. This approach enabled the resulting data analysis to identify top priorities for changes in TA practice and professional development but did not allow a more nuanced look at secondary expectations. Studying how many expectations and in what order of priority students place those expectations would require a more narrow and focused study on TAs and is a topic for future work.

Finally, this study design is not longitudinal. Each individual participant’s data represents a single point in time. Thus, the study illuminates little about how engineering students’ views of TA evolve over time as they become increasingly acclimated to their home (engineering) department’s culture and ways of doing things. A large majority of the data in this population was collected from sophomore or junior-level classes, however, and gives important indications regarding what students expect and need from TAs as they are entering into their engineering major and chosen field of study.

## Conclusions

Student expectations for TAs from a broad range of mechanical and electrical and computer engineering courses involving students from multiple engineering and physics majors have been investigated using thematic analysis of qualitative (text-based) data and quantitative analysis of results to evaluate statistical significance. Results indicate that the largest proportion of students expect TAs to provide high-quality interactions with students, specifically with respect to frequent contact and opportunities for active learning. Students also expect that their TAs are prepared, specifically with regard to high-quality presentation of content and accompanying guidance on how to apply that content to engineering problems. A small but important percentage of students also expect that TAs improve their hospitality by being more welcoming, friendly, flexible, and caring.

Differences by gender, race/ethnicity, and international status emerged in student expectations for TAs in this study, indicating that TA practices should not only focus on what the majority of students want, but also consider specifically what is different about the expectations of underrepresented groups, including international students. Given the changing dynamics of student expectations during different time periods and across demographic groups, future work will focus on developing and implementing a diagnostic tool that clarifies expectations at the outset of each course or academic year and provides guidelines to TAs for tailoring their practices to those expectations. While meeting student expectations will likely have a significant impact on student satisfaction, these efforts must also be blended with principles of excellent pedagogy. In particular, TAs can and should play an important role in making learning more active and student-centered, by focusing on high-quality interactions, preparing high-quality content and guidance, and expressing care and hospitality to students.
